# Total humerus replacement for osteosarcoma with proximal part of humerus: a case report

**DOI:** 10.1186/1477-7819-10-36

**Published:** 2012-02-14

**Authors:** Yukihiro Yoshida, Yasuaki Tokuhashi

**Affiliations:** 1 Department of Orthopedic Surgery, Nihon University School of Medicine, 30-1, Oyaguchikami-cho, Itabashi-ku, Tokyo 173-8610, Japan

**Keywords:** Total humerus replacement, Osteosarcoma

## Abstract

Incisional biopsy and intramedullary pinning were performed for pathological fracture associated with a malignant bone tumor of the proximal humerus. Osteosarcoma, for which preoperative chemotherapy had been performed, was confirmed by postoperative pathological examination. To achieve wide resection and acquire a safe resected margin, total humerus replacement was performed, and the whole humerus was reconstructed using the Howmedica Modular Reconstruction system. The patient resumed normal activities, although mild contracture of the elbow joint remains 8 years after surgery.

## Background

Total humerus replacement following resection of a malignant bone tumor of the upper limbs is a rarely performed procedure [1]. We performed open reduction and internal fixation simultaneously with incisional biopsy in a patient with a bone tumor of the humerus, pathological fracture of which had been caused by a metastatic bone tumor or the primary malignant bone tumor. Osteosarcoma was diagnosed, and total humerus replacement was performed after chemotherapy. Preservation of the affected limb was successful. In the 8 years, to date, since surgery the patient has remained recurrence-free. Herein, we present this case in details.

## Case presentation

The patient was a 48-year-old male with a chief complaint of right upper arm pain. His past medical and familial histories were unremarkable. On February 5, 2002, severe pain occurred in the right upper arm while exercising with an iron bar, and he visited a physician. The patient was referred and admitted to our department for suspected pathological fracture associated with a metastatic bone tumor on February 20. Swelling and local heat were present in the proximal region of the right forearm, and tenderness and pain during motion were also present. No spontaneous pain was observed. The range of motion of the right shoulder joint could not be measured because of pain. On admission, alkalinephosphatase, at 442 and C-reactive protein at 1.05, were both markedly elevated. All tumor markers were negative, and there were no abnormal findings on other tests.

**Figure 1:**
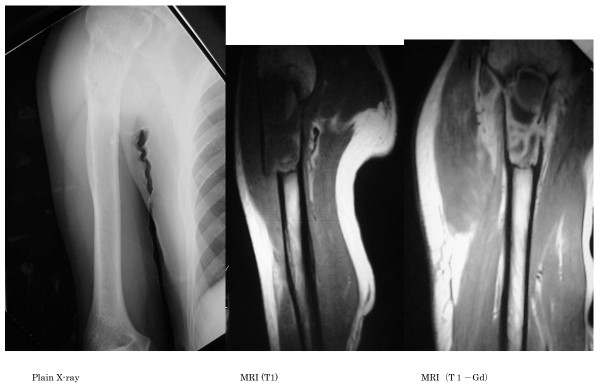
**Plain X-ray radiographs and MRI on the first examination**. Osteolytic hypertranslucency accompanied by pathological fracture was observed in the proximal humerus on plain radiographs. The periosteal reaction was not severe. On MRI, low intensity was detected on T1-weighted imaging, while cyst-like changes and low intensity of the tumor content were seen on T1 gadolinium contrast imaging.

**Figure 2:**
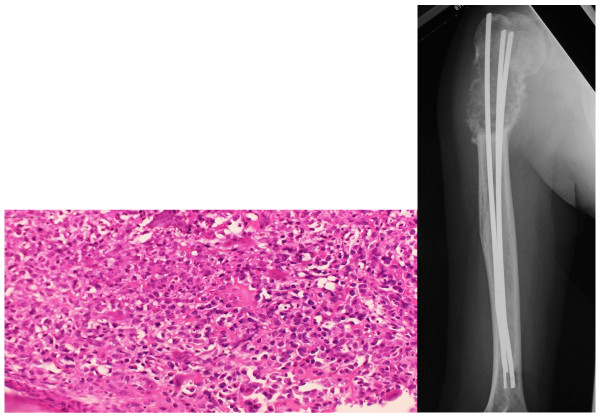
**Histopathology on biopsy and plain radiographics image at the completion of preoperative chemotherapy**. Immature bone tissue and osteoid were not reactive, and were judged to be tumorous. Osteoblastic-type osteosarcoma was ultimately diagnosed. Chemotherapy was judged to have achieved a complete response (CR).

On radiographs obtained at the first examination, a pathological fracture was noted in the proximal humerus, showing hypertranslucency of bone, mainly involving osteolytic changes (Figure 1). On computed tomography (CT), the bone cortex was thinned, and showed a relatively homogenous structure. On magnetic resonance imaging (MRI), a low intensity was observed on T1-weighted imaging, while the fractured region was mainly enhanced on T1 gadolinium contrast imaging, and high intensity was observed on T2-weighted imaging (Figure 2). Bone scintigraphy showed signal accumulation in the proximal humerus. Based on these findings, pathological fracture associated with a metastatic bone tumor or primary malignant bone tumor in the right proximal humerus was diagnosed, and incisional biopsy and open reduction and internal fixation (ORIF) were performed on February 25, 2002. The treatment consisted of three Ender's pins inserted at the posterior aspect of the elbow joint. Swelling of the right proximal upper arm became more severe after treatment. On pathological examination, tumorous immature bone tissue and osteoid were present; based on these findings, an osteoblastic-type sarcoma was diagnosed.

**Figure 3:**
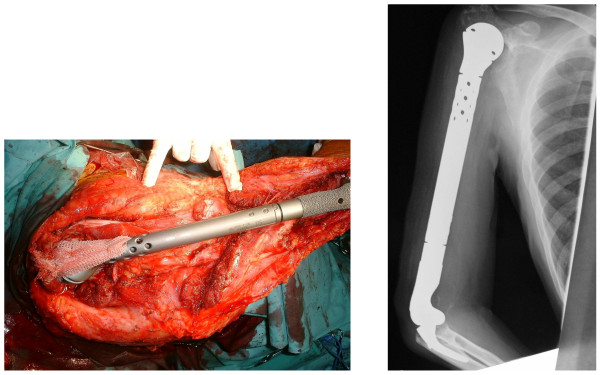
**Intraoperative findings and a postoperative plain radiographics images**. Total humerus replacement was performed, and an HMRS artificial joint for tumor treatment was inserted and hung using an artificial ligament. We used the Leeds-Keio as the artificial ligament for this procedures. The ISOLS radiographics evaluation was excellent for all items.

After about 4 months of preoperative chemotherapy for osteosarcoma, sclerotic changes centered within the area of bone hypertranslucency were observed on plain X-ray and CT images. In addition, the accumulation on bone scintigraphy disappeared. After preoperative chemotherapy had been judged to have achieved a complete response (CR), massive extra-capsular resection of the shoulder joint was performed, followed by total humerus placement on July 3, 2002. The humerus was entirely resected including parts of the deltoid and triceps brachii muscles and cuff, whereas the long head of the biceps brachii muscle, musculocutaneous nerve, and other neurovascular bundles were conserved. In reconstruction of the cuff, an artificial ligament was attached around an artificial bone head for tumor treatment, and the artificial joint was entirely covered with a latissimus dorsi muscle flap. In the postoperative specimen, the lesion was completely and widely resected. Histopathologically, the lesion was mostly necrotized, being close to Grade 3 employing the tissue judgment scale established by the Musculoskeletal Tumor Committee of the Japanese Orthopaedic Association (Figure 3). Postoperative chemotherapy was discontinued because of severe liver dysfunction. As of 8 years after surgery, the patient's condition has remained favorable, with no local recurrence or metastasis. Abduction, flexion and extension of the shoulder joint were 10, 20 and 5°, respectively, external and internal rotations were -20 and 90°, respectively, and flexion at the elbow joint was 20-120°. The strengths of the biceps and triceps brachii muscles were 4 and 2, respectively. The Enneking functional score was 80%: pain, 5; function, 3; emotional acceptance, 3; hand positioning, 3; manual dexterity, 5; and lifting ability, 3. On the International Society of Limb Salvage X-ray evaluation, all items of bone remodeling, interface, and anchorage were excellent.

## Discussion

Osteosarcoma is a malignant tumor forming tumorous bone and cartilage or osteoid. In the nationwide bone tumor registry established by the Musculoskeletal Tumor Committee of the Japanese Orthopaedic Association, 7,649 of a total of 49,768 bone tumors registered from 1972 to 2003 (32 years) were malignant (15%); 3256 of these were osteosarcomas (accounting for 42% of bone malignances). Development at 40 years of age or older is reportedly rare, but 433 (13.2%) of the 3,256 osteosarcoma patients were 40 years of age or older. Regarding the developmental site, osteosarcoma most frequently arises in the femur and tibia, followed by the humerus in 262 cases (8%), being ranked the 3^rd^[1].

It is stated in Dahlin's Bone Tumors that the incidence in those in their 40s is about 5%, and that osteosarcoma developed in the humerus in 154 of 1952 cases, accounting for 7.8% of all osteosarcoma cases [2].

For the differential diagnosis from metastatic bone tumor, which was a challenge in our present patient, a comprehensive judgment should be based on past medical history, age, leison, and imaging findings. In patients 60 years of age and older, cancer metastasis is initially suspected. The incidence of metastatic cancer is high in the spine and pelvis, and the periosteal reaction on plain radiographs is poor in a metastatic bone tumor. The primary lesion should also be simultaneously investigated. In our present patient, bone destruction was marked, and the periosteal reaction was poor. Arterial bone cyst, giant cell tumor of bone, conventional osteosarcoma, and telangiectatic osteosarcoma are included in the differential diagnosis. Aneurysmal bone cyst was inconsistent with age, and the developmental site was not typical for giant cell tumor of bone [3]. Osteosarcoma, particularly, telangiectatic osteosarcoma, was strongly suspected on imaging, but a metastatic bone tumor was also considered based on the patient's age and the higher prevalence of this type of tumor. Intramedullary pinning was selected because plate fixation was difficult due to the presence of pathological fracture and the location of the lesion in the proximal humerus.

The challenge faced by surgeons after the resection of a malignant bone tumor in the proximal humerus is reconstruction of shoulder joint function. When the cuff is included in massive resection to acquire a safe resection margin, shoulder joint function is mostly lost. For reconstruction of the proximal end of the humerus, several methods employing an artificial bone head for tumor treatment [4], [5], allogeneic bone grafting [6], [7], processed bone [8], and vascularized bone grafting [9], [10]have been reported, but reconstruction by hanging these from the acromion using an artificial ligament, fascia, or tendon is inevitably necessary. We considered the distal humerus to be contaminated with tumorcomponents via intramedullary pinning, and adopted total humerus replacement to ensure the safety of the resection margin.

For the proximal humerus, hanging from the acromion using an artificial ligament was adopted. Our resection method was corresponded closely with that for type V of the Malawer classification of proximal humeral malignant bone tumors, but we opted to perform massive resection corresponding to extra-capsular resection conserving the acromion and coracoid process because chemotherapy had been sufficiently effective [11]. Very few cases of total humerus replacement have been reported. To our knowledge, only Grimer (1996) [12] and Fabroni (1999) [4] have reported such cases and Ayoub (1999) reported described extendable artificial joint use for the humerus [13]. Fabroni et al. reported long-term outcomes in 3 cases, all receiving custom-made artificial joints, and the mean functional score was about 65%. The characteristics of the Howmedica Modular Reconstruction System (HMRS) for the humerus used herein include the adjustability of the stem length corresponding to the resected bone mass and the presence of a hole for attachment with soft tissue. In addition, a porous coating is applied, and the ulnar component of the elbow joint has an anatomical shape. However, the level of osteotomy for optimal setting of the ulnar component of the elbow joint is unclear, and improvement may be necessary.

## Conclusion

A pathological fracture due to a metastatic bone tumor of the proximal humerus was initially diagnosed. Intramedullary pinning was performed. However, osteosarcoma was confirmed by postoperative pathological examinations. Therefore, preoperative chemotherapy was conducted, followed by wide resection to acquire a safe margin, and total humerus replacement was then performed. The HMRS was used for reconstruction of the whole humerus. The patient resumed normal activities, although mild contracture of the elbow joint remains 8 years after surgery. In Japan, HMRS is the only artificial joint for tumor treatment that covered National Health Insurance. Reconstruction of the whole humerus using HMRS is rare, but this reconstruction method is an options, by which a favorable limb function can be achieved.

## Consent

Written informed consent was obtained from the patient for publication of this Case report and any accompanying images. A copy of the written consent is available for review by the Editor-in-Chief of this journal.

## List of abbreviations

CT: Computed tomography; MRI: Magnetic resonance imaging; ORIF: Open reduction and internal fixation; CR: Complete response; HMRS: Howmedica Modular Reconstruction System

## Competing interests

The authors declare that they have no competing interests.

## Authors' contributions

YY contributed to collection of the clinical data and writing of the manuscript. YT contributed to the writing and editing of the manuscript. Both authors read and approved the final manuscript.

## References

[B1] JOA Musculoskeletal tumor committee. Bone Tumor Registry in Japan National Cancer Center 2008

[B2] Unni KK Inwards CY Dahlin's Bone Tumors Lippincott Wilianms&Wilikins 2010 Sixth22207540

[B3] Yarmish G Klein MJ Landa J Lefkowitz RA Hwang S Imaging characteristics of primary osteosarcoma: nonconventional subtypes Radiographics 2010 30 6 1653 672 10.1148/rg.306105524 21071381

[B4] Fabroni RH Castago A Aguilera AL Steverlynck AM Zeballous J Long -term results of limb salvage with the Fabroni custom made endoprosthesis Clin Orhtop Relat Res 1999 35 8 41 52 9973975

[B5] Ross AC Sneath RS Scales JT Endoprosthetic replacement of the humerus and elbow joint J Bone Joint Surg [Br] 1987 69-B 652 55 10.1302/0301-620X.69B4.3611176 3611176

[B6] Mankin HJ Doppelt SH Sullivan TR Tomford WW Osteoarticular and intercalary allograft transplantation in the management of malignant tumors of bone Cancer 1982 50 613 30 10.1002/1097-0142(19820815)50:4<613::AID-CNCR2820500402>3.0.CO;2-L 7046906

[B7] Mankin HJ Gebhardt MC Jennings LC Springfield DS Tomford WW Long-term results of allograft replacement in the management of bone tumors Clin Orthop Relat Res 324 1996 86 97 10.1097/00003086-199603000-00011 8595781

[B8] Muramatsu K Fukano R Iwanaga R Taguchi T Reconstruction of the proximal humerus by combined use of extracorporeally-irradiated osteochondral graft and free vascularized fibula following resection of Ewing sarcoma Journal of Plastic, Reconstructive &Aesthetic Surgery 2010 63 2177 180 10.1016/j.bjps.2010.03.008 20347410

[B9] Wada T Usui K Isu K Yamawakii S Ishii S Reconstruction and limb salvage after resection for malignant bone tumour of the proximal humerus. A single procedure using a free vasculaized fibular graft J Bone Join Surg 1999 81B 808 13 10.1302/0301-620x.81b5.9430 10530841

[B10] Rose PS Shin AY Bishop AT Moran SL Sim FH Vascularized free fubula transfer for oncogenic reconstruction of the humerus Clin Orthop Relat Res 2005 438 80 4 16131873 10.1097/01.blo.0000179586.34727.5b

[B11] Bickels J Wittig JC Kollender Y Kellar-Graney K Meller I Malawer MM Limb-sparing resections of the shoulder girdle J Am Coll Surg 2002 194 4 422 35 10.1016/S1072-7515(02)01124-9 11949748

[B12] Grimer JG Carter SR sneath SR Manegement of a huge tumour of the humerus by total replacement of the humerus:an 11-year foolow up Arch Orthop Trauma Surg 1998 117 298 99 10.1007/s004020050253 9581268

[B13] Ayoub KS Fiorenza F Grimer RJ Tilman SR Carter RS Extendable endoprostheses of the humerus after resection of bone tumours J Bone Joint Surg [Br] 1999 81-B 495 500 10.1302/0301-620x.81b3.9178 10872374

